# Fellow-Workers and Their Doings

**Published:** 1914-03-21

**Authors:** 


### ROUND THE WORLD.

### Fellow-Workers and their Doings.

[The Editor Invites Early Information and Contributions Marked "Fellow-Workers."]

Mr. W. R. Ransford, L.D.S. Eng., who has been appointed dental surgeon to the General Hospital at Rangoon and lecturer in dental surgery at the Burma Government Medical School, is an old Guy's Hospital man and qualified from the well-known dental school there in 1903.

Dr. Edward Mellanby, M.A., M.B., the lecturer on physiology at St. Thomas's Hospital, who is a Beit Memorial Fellow, is concluding a research on the toxins which cause the profound collapse and intoxication in cases of enzymic diarrhœa, a research which was instituted at the instigation of the Local Government Board.

Dr. Charles Singer, M.D., M.R.C.P., who has just been awarded the Philip Walker studentship in pathology of Oxford University, pursued his studies at Magdalen College before proceeding to St. Mary's Hospital, where he won several important prizes, and held various resident appointments. During the last few years Dr. Singer has carried out pathological researches at the Cancer Hospital and abroad; he has also made several interesting communications to various learned societies dealing with the history of medicine.

Mr. H. D. Gillies, F.R.C.S., the new chief assistant in the throat department at St. Bartholomew's Hospital, has also been appointed aural surgeon to the Alexandra Hospital for Children. Mr. Gillies, who had a distinguished career as a student at "Bart.'s," where, amongst other distinctions, he won the Luther Holden Research Scholarship, is surgeon to the throat, nose, and ear department at the Prince of Wales's General Hospital, Tottenham. He is also one of the best amateur golfers in England.

The recently appointed physician in charge of the $x$-ray and electro-therapeutic departments at St. John's Hospital for Diseases of the Skin in Leicester Square, Mr. Christopher R. Kempster, has had wide experience in various departments of medical work. Apart from his interests in radiography and electricity, Mr. Kempster is a Fellow of the Society of Medical Officers of Health, and was formally civil surgeon to the War Office and assistant divisional surgeon to the Metropolitan Police.

Dr. Murtaugh James Houghton, M.R.C.S., of Ilford, has recently invented an appliance which not only greatly increases the heating power but also lessens the coal consumption of ordinary fire grates. Dr. Houghton is a man of many activities; amongst other things, he is a medical referee under the Workmen's Compensation Act, whilst the Ilford Hospital is the product of his continued personal labours. An agitation that he set on foot resulted in the establishment of a local municipal ambulance service that is always available.

Physiology is also represented by another candidate for the coveted F.R.S—Dr. Henry Hallett Dale, who is the director of the Wellcome Physiological Research Laboratories at Herne Hill. A former student of St. Bartholomew's Hospital, Dr. Dale became an M.D. Cantab. in 1909. He has contributed numerous important communications to scientific societies, both in regard to physiological and pharmacological researches.

Dr. Frank Heasman, M.R.C.S., L.R.C.P., who has just been promoted to the post of physician to the Royal Victoria and West Hants Hospital at Bournemouth, is an old "Bart.'s" man, and has been in practice at Boscombe for some years.

Dr. E. G. Fearnsides, M.B., M.R.C.P., assistant physician to the Maida Vale Hospital for the Paralysed and Epileptic, has just concluded a research into the clinical features of the outbreaks of polio-myelitis which have occurred in London during the last two years. His observations tend to show how the cases may present symptoms ranging from meningitis or local palsies to sudden death, and that in any one case the lesions are more widespread than is usually thought. Before coming to London Dr. Fearnsides was well known in Cambridge as a parasitologist.

In these days of pessimistic regard for slackness of intestinal activity, and tendency to remove important organs such as the colon when function becomes impaired, it is refreshing to find that Dr. Francis Hernaman-Johnson, of Darlington, can report so satisfactorily on the electrical treatment of intestinal stasis (*British Medical Journal*, March 7). According to him, careful treatment with either sinusoidal or static electricity will relieve all mild cases of this troublesome disorder, and at least half the bad cases. This is a very different thing from a serious surgical operation necessitating prolonged rest in bed. Dr. Hernaman-Johnson is an old "Bart.'s" man and a well-known provincial practitioner, who has been for some years physician to the $x$-ray and electrical departments at Darlington General Hospital; he is an M.D. of Aberdeen University, where he had a brilliant academic career before taking up hospital work.

The third medical candidate, Dr. Arthur Edwin Boycott, is professor of pathology at Manchester University. His medical education was obtained at Oxford and St. Thomas's. Formerly a lecturer on pathology at Guy's Hospital, Dr. Boycott is well known for various brilliant researches in his special subject.

Dr. E. Curtin, of Bournemouth, has been directing attention to the important matter of attempting to estimate the exact value which can be attached to different methods of treating acute pneumonia. So much confidence is placed in the ice-pack, venesection, and strychnine, used largely on empirical grounds, that an analysis of the subject from the strictly physiological and pathological points of view is of importance to every practitioner. Dr. Curtin, who is a keen clinical observer, in collating his observations (*Practitioner*, March 1914), points out that the ice-pack applied indiscriminately to the chest must really do more harm than good in the majority of cases; whilst he considers that blood-letting is likely to lower the patient's strength seriously without having any compensating advantage. Thirdly, he finds that strychnine cannot be of the slightest real use, a conclusion supported by the now accepted fact that it has no direct action as a heart stimulant. On the other hand, this observer thinks that ice may be applied with advantage to the neck and head.

